# Differences in survival prognosticators between children and adults with H3K27M‐mutant diffuse midline glioma

**DOI:** 10.1111/cns.14307

**Published:** 2023-06-13

**Authors:** Xuan Gong, Shuwen Kuang, Dongfeng Deng, Jun Wu, Longbo Zhang, Chao Liu

**Affiliations:** ^1^ Departments of Neurosurgery, Xiangya Hospital Central South University Changsha China; ^2^ National Clinical Research Center for Geriatric Disorders Xiangya Hospital, Central South University Changsha China; ^3^ Departments of Oncology Xiangya Hospital, Central South University Changsha China

**Keywords:** adults, age, children, diffuse midline glioma, H3K27M‐mutant, prognosis

## Abstract

**Aims:**

H3K27M‐mutant diffuse midline glioma (DMG) is a rare and aggressive central nervous system tumor. The biological behavior, clinicopathological characteristics, and prognostic factors of DMG have not yet been completely uncovered, especially in adult patients. This study aims to investigate the clinicopathological characteristics and identify prognostic factors of H3K27M‐mutant DMG in pediatric and adult patients, respectively.

**Methods:**

A total of 171 patients with H3K27M‐mutant DMG were included in the study. The clinicopathological characteristics of the patients were analyzed and stratified based on age. The Cox proportional hazard model was used to determine the independent prognostic factors in pediatric and adult subgroups.

**Results:**

The median overall survival (OS) for the entire cohort was 9.0 months. Significant differences were found in some clinicopathological characteristics between children and adults. The median OS was also significantly different between the pediatric and adult subgroups, with 7.1 months for children and 12.3 months for adults (*p* < 0.001). In the overall population, the multivariate analysis identified adult patients, single lesion, concurrent chemoradiotherapy/radiotherapy, and intact ATRX expression as independent favorable prognostic factors. In the age‐stratified subgroups, the prognostic factors varied between children and adults, with intact ATRX expression and single lesion being independent favorable prognostic factors in adults, while infratentorial localization was significantly associated with worse prognosis in children.

**Conclusions:**

The differences in clinicopathological features and prognostic factors between pediatric and adult patients with H3K27M‐mutant DMG suggest the need for further clinical and molecular stratification based on age.

## Graphical abstract

H3K27M‐mutant diffuse midline glioma (DMG) is a heterogeneous type of gliomas with distinct clinicopathological features and showed a significant difference in overall survival between children and adults. Notably, there were differences in prognostic factors between them. Our results suggested DMG should be clinically and molecularly segregated according to different age.

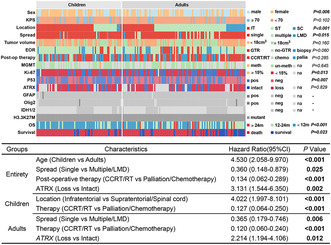

## 1 INTRODUCTION

With the development of molecular techniques and diagnoses, studies have found that K27M mutations in H3 histone encoded by H3F3A (H3.3) or HIST1H3B/C (H3.1) are associated with a worse prognosis in diffuse intrinsic pontine glioma (DIPG).^1^, ^2^ The 2016 WHO classification of central nervous system (CNS) tumors first proposed “diffuse midline glioma, H3K27M‐mutant” (H3K27M‐mutant DMG),^3^ which is characterized by the midline location in CNS with histological characteristics of glioma, diffuse and infiltrative growth pattern, and H3K27M mutation.

H3K27M‐mutant DMG is a rare cranial malignancy, with an estimated 200 to 300 new pediatric patients diagnosed annually in the United States.^4^ Previous studies have shown that H3K27M‐mutant DMG is more commonly observed in children and adolescents, with the peak incidence occurring among 3–10 years of age and a similar distribution between sexes.^5^ Conversely, this type of tumor occurs less frequently in adults.^6^ Consequently, the clinicopathological characteristics, treatment strategies, and prognostic factors of DMG remain incompletely understood, particularly in the adult population.

The distinctive molecular feature of DMG is the presence of H3K27M mutations in H3.3 or H3.1 histones. Consensus genetic findings indicate that mutations in isocitrate dehydrogenase (*IDH*), promoter methylation of O(6)‐methylguanine‐DNA methyltransferase (*MGMT*), and amplification of epidermal growth factor receptor (*EGFR*) are mutually exclusive with H3K27M mutation. On the other hand, mutations in *P53* and amplification of platelet‐derived growth factor receptor A (*PDGFRA*) often co‐occur with H3K27M mutation and impact the prognosis of DMG.^7^, ^8^, ^9^ Additionally, several other gene alterations, including activin A receptor type I (*ACVR1*), fibroblast growth factor receptor 1 (*FGFR1*), *BRAFV600E*, and alpha‐thalassemia mental retardation X‐linked (*ATRX*) mutations, have been associated with the survival of DMG.^7^, ^8^, ^10^, ^11^ However, the molecular alterations in H3K27M‐mutant DMG are poorly comprehended, and their prognostic significance remains unknown.

The conventional treatment options for H3K27M‐mutant DMG include surgery and postoperative adjuvant therapy, such as radiotherapy and chemotherapy.^12^, ^13^ However, due to the deep location, infiltrative and diffuse growth pattern of DMG, complete surgical resection is often impractical, particularly for tumors located in the brainstem. This is further complicated by the high risk of serious postoperative deficits.^14^ Therefore, the decision to perform resection or biopsy should be carefully considered, weighing the potential benefits of extent of operation against the risks of postoperative complications. Radiotherapy has shown improvements in both quality of life and prognosis for patients with DMG.^15^, ^16^, ^17^, ^18^ However, the addition of temozolomide (TMZ) to radiotherapy remains controversial owing to the specific genetic characteristic of *MGMT* promoter unmethylation and intact blood–brain barrier.^19^, ^20^, ^21^

Despite undergoing complex clinical management, including surgical interventions and adjuvant therapies, the median overall survival (OS) for pediatric patients with H3K27M‐mutant DMG ranges from 9 to 12 months, with a 2‐year OS of less than 10% and a 5‐year OS of less than 1%.^22^, ^23^, ^24^ It is important to note that most survival data available for H3K27M‐mutant DMG are derived from children with DIPG, only representing a DMG subgroup. Comparative survival data between adults and children within the same study are limited and show inconsistent results. Some retrospective studies have reported significantly longer OS in adults compared to the pediatric group,^25^, ^26^, ^27^ while others have found similar prognoses between adults and children.^28^, ^29^ There is also a scarcity of studies examining prognostic factors between pediatric and adult subgroups.

In this single‐center large cohort study of 171 patients with H3K27M‐mutant DMG, one of the largest sample sizes to date, we presented the clinical and pathological characteristics of the entire population and subgroups stratified by age. We conducted univariable and multivariable analyses on the entire cohort and age‐stratified subgroups to identify factors impacting survival, respectively.

## 2 MATERIALS AND METHODS

### 2.1 Patient selection

In this retrospective single‐center study, H3K27M‐mutant DMG patients who were diagnosed and treated at Xiangya Hospital of Central South University in China between October 2016 and January 2022 were reviewed. Two experienced neuropathologists reviewed the histopathologic findings according to the 2016 WHO classification of CNS tumors.^3^

The inclusion criteria for this study were as follows: (1) pathologic diagnosis of H3K27M‐mutant DMG based on the 2016 WHO classification of CNS tumors, including tumors located in midline structures of CNS with histological characteristics of glioma, diffuse and infiltrative growth pattern, and confirmed H3K27M mutation through immunohistochemistry (IHC) and Sanger sequencing; (2) newly diagnosed DMG with the first operation or biopsy; and (3) availability of complete follow‐up materials (see “Data Collection” section for details). The exclusion criteria were as follows: (1) patients with ganglioglioma or ependymoma with H3K27M mutation; (2) patients who had relapsed DMG or received chemotherapy, radiotherapy, or targeted therapy before the pathologically confirmed diagnosis of H3K27M‐mutant DMG.

Based on the inclusion and exclusion criteria, 24 patients were excluded, and the final analysis included 171 patients. There was one patient having a previous diffuse astrocytoma (WHO grade II) diagnosis in 2014 before the recognition of DMG. This patient underwent a second operation in 2016 because of local recurrence and was subsequently diagnosed with H3K27M‐mutant DMG. We re‐examined the tissue sample from the first operation and confirmed the presence of H3K27M mutation. Therefore, this patient was also included in our study. The study protocol was approved by the Ethics Committee of Xiangya Hospital, Central South University. Informed consent was obtained from each patient or their legal guardian, and all patient data were treated with confidentiality.

### 2.2 Data collection

In this study, we collected the following data for each patient: general data including gender, age, and Karnofsky Performance Score (KPS); radiographic data including preoperative, postoperative, and follow‐up magnetic resonance (MR) imaging; extent of surgical resection; and postoperative treatments such as radiotherapy, chemotherapy, and palliative treatment. The evaluation criteria of extent of surgical resection were as follows: Based on postoperative imaging, gross total resection (GTR) was defined as a removal of >95% of the tumor mass; non‐GTR included subtotal resection and partial resection of craniotomy.^30^ The radiological features assessed included tumor location, number of tumors, tumor volume (calculated based on maximal diameters), and presence of leptomeningeal spreading (LMD). LMD was characterized with anomalous enhancement of the brain surface, cerebellar foliae, cerebral sulci, cranial nerves, or spinal nerve roots on MR imaging.

Primary antibody used for histopathology and IHC in this study are listed as follows: H3K27M (RMA‐0840, MXB Biotechnologies), Ki‐67 (RMA‐0731, MXB Biotechnologies), P53 (MAB‐0674, MXB Biotechnologies), ATRX (MAB‐0855, MXB Biotechnologies), GFAP (MAB‐0769, MXB Biotechnologies), Olig2 (RMA‐0681, MXB Biotechnologies), MGMT (MAB‐0361, MXB Biotechnologies), and IDH1 (MAB‐0733, MXB Biotechnologies). For H3K27M, tumor cells nuclear staining was determined as positive and no nuclear staining was determined as negative. For Ki‐67, the index was calculated as a percentage of positively stained nuclear by 1000 tumor cells counted under 400× magnification in the areas with highest density of positive nuclear. For P53, >30% of nuclear staining was the evidence of *TP53* mutation and interpreted as P53 positive. For ATRX, no nuclear staining was considered as evidence of loss. For Olig2 and GFAP, >50% of nuclear and cytoplasmic staining were interpreted as positive, respectively. For IDH1, cytoplasmic staining was interpreted as positive. For MGMT, nuclear staining was determined as MGMT promoter unmethylation. Molecular detections were also conducted to investigate *MGMT* promoter methylation, H3.3K27M mutation, and *IDH* mutation. Sanger sequencing was used for *IDH1/2* and H3.3K27M mutation analysis, and methylation‐specific quantitative PCR was employed to determine the status of *MGMT* promoter methylation.

### 2.3 Statistical analysis

The patient baseline data were analyzed by direct counting, and non‐normally distributed measurement data were reported as medians. Data processing was conducted using GraphPad Prism 8 (GraphPad Software) and SPSS 23.0. Statistical analyses included the Student *t*‐test or Chi‐square (Fisher exact) test as appropriate. The starting point for OS analysis in DMG patients was the time of the first operation, and the endpoint was either death or the last follow‐up date. The study's follow‐up period ended in June 2022. OS was assessed using Kaplan–Meier survival curves and compared using the log‐rank test. Multivariate survival analysis was performed using the Cox proportional hazard model. *p*‐Value < 0.05 was considered statistically significant.

## 3 RESULTS

### 3.1 Clinical and pathological characteristics of 171 patients with H3K27M‐mutant DMG and subgroups stratified by age

A total of 171 patients with H3K27M‐mutant DMG were included in the study. Among them, 69 (40.4%) cases were children (age below 18 years) and 102 (59.6%) cases were adults. Tumors were anatomically located in the infratentorial region (brainstem, *n* = 74), spinal cord (*n* = 19) and supratentorial region including the thalamus (*n* = 53), the basal ganglia (*n* = 11), the ventricles (*n* = 4), corpus callosum (*n* = 5), pineal gland (*n* = 3), and suprasellar region (*n* = 2). Majority of the DMG located in the infratentorial region in children (68.1%), while it more frequently occurred in the supratentorial region in adults (57.8%) (*p* < 0.001). In our series, 146 patients (85.4%) underwent surgical resection, including 15.8% with GTR and 69.6% with non‐GTR, while 25 patients (14.6%) underwent stereotactic biopsy. After surgery, the majority of patients (*n* = 89, 52.0%) received either concurrent chemoradiotherapy (CCRT) or radiotherapy alone (RT), while 82 patients (48.0%) chose palliation therapy or chemotherapy alone. The median follow‐up duration was 14.6 months (range, 5.2–93.1 months). At the final follow‐up, only 43 patients (25.1%) were still alive, including 11 in the pediatric subgroup and 32 in the adult subgroup. All alive pediatric patients underwent postoperative CCRT; two patients achieved GTR and nine received non‐GTR. Among the alive adult patients, 27 patients received CCRT, one underwent chemotherapy alone, and four chose palliative treatment; as for extent of surgical resection, six with GTR, 22 with non‐GTR, and four with stereotactic biopsy.

**FIGURE 1:**
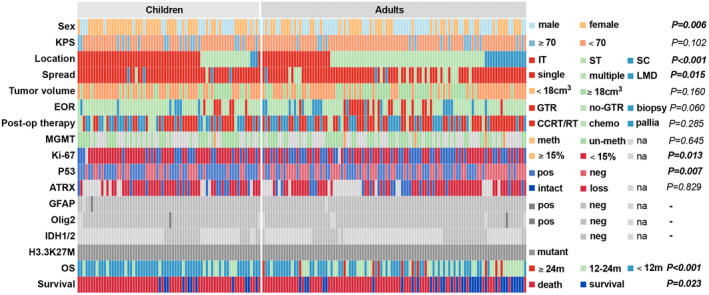
Schematic summary of clinicopathological characteristics in H3K27M‐mutant DMGs. ATRX, alpha‐thalassemia mental retardation X‐linked; CCRT/RT, concurrent chemoradiotherapy/radiotherapy; chemo, chemotherapy; EOR, extent of resection; GFAP, glial fibrillary acidic protein; GTR, gross total resection; IDH1/2, isocitrate dehydrogenase 1/2; IT, infratentorial tumor; KPS, Karnofsky performance status; LMD, leptomeningeal dissemination; m, months; meth, methylated; MGMT, O(6)‐methylguanine‐DNA methyltransferase; na, not available; neg, negative; Olig2, oligodendrocyte transcription factor 2; OS, overall survival; pallia, palliation; pos, positive; SC, spinal cord; ST, supratentorial tumor.

H3.3K27M mutation was confirmed in tumor tissue from all 171 patients using IHC or Sanger sequencing. Based on Ki‐67 staining, pediatric patients showed a higher proliferative index compared to adults (71.6% vs. 51.5%, *p* = 0.013). Furthermore, positive P53 status was more frequently observed in children than in adults (65.2% vs. 44.1%, *p* = 0.007). The loss of ATRX expression was uncommon in DMG (26.9%), and there was no significant difference between children and adults. The clinical, histologic, and molecular characteristics are summarized in Table 1 and Figure 1.

**TABLE 1:** Demographic characteristics of H3K27M‐mutant DMG patients in the entire cohort and stratified by age.

Characteristics	No. of patients (%)			
	Total	Children	Adults	*p*‐Value
No. of patients	171	69 (40.4)	102 (59.6)	
Mean age (years)	27.0 ± 18.3	8.6 ± 3.9	39.4 ± 12.8	
Sex				
Male	79 (46.2)	23 (33.3)	56 (54.9)	**0.006** ^*^
Female	92 (53.8)	46 (66.7)	46 (45.1)	
KPS				
KPS ≥ 70	139 (81.3)	52 (75.4)	87 (85.3)	0.102
KPS < 70	32 (18.7)	17 (24.6)	15 (14.7)	
Location				
Infratentorial	74 (43.3)	47 (68.1)	27 (26.5)	**<0.001** ^*^
Supratentorial	78 (45.6)	19 (27.5)	59 (57.8)	
Spinal cord	19 (11.1)	3 (4.4)	16 (15.7)	
Spread				
Single lesion	145 (84.8)	63 (91.3)	82 (80.4)	**0.015** ^*^
Multiple lesions	19 (11.1)	2 (2.9)	17 (16.7)	
LMD	7 (4.1)	4 (5.8)	3 (2.9)	
Tumor volume (cm^3^)^a^				
<Median	98 (57.3)	44 (63.8)	54 (52.9)	0.160
≥Median	73 (42.7)	25 (36.2)	48 (47.1)	
Extent of resection				
GTR	27 (15.8)	7 (10.1)	20 (19.6)	0.060
Non‐GTR	119 (69.6)	55 (79.8)	64 (62.8)	
Biopsy	25 (14.6)	7 (10.1)	18 (17.6)	
Postoperative therapy				
CCRT/RT	89 (52.0)	32 (46.4)	57 (55.9)	0.285
Chemotherapy alone	16 (9.4)	9 (13.0)	7 (6.9)	
Palliation	66 (38.6)	28 (40.6)	38 (37.2)	
*MGMT* promoter				
Methylated	5 (5.5)	1 (2.8)	4 (7.3)	0.645
Unmethylated	86 (94.5)	35 (97.2)	51 (92.7)	
Ki‐67 index				
≥15%	100 (59.2)	48 (71.6)	52 (51.5)	**0.013** ^*^
<15%	69 (40.8)	20 (29.4)	49 (48.5)	
P53				
Positive	90 (52.6)	45 (65.2)	45 (44.1)	**0.007** ^*^
Negative	81 (47.4)	24 (34.8)	57 (55.9)	
ATRX				
Intact	95 (73.1)	40 (74.1)	55 (72.4)	0.829
Loss	35 (26.9)	14 (25.9)	21 (27.6)	
GFAP				
Positive	168 (99.4)	67 (98.5)	101 (100.0)	
Negative	1 (0.6)	1 (1.5)	0 (0.0)	
Olig2				
Positive	161 (98.8)	66 (98.5)	95 (99.0)	
Negative	2 (1.2)	1 (1.5)	1 (1.0)	
*IDH1/2*				
Positive	0 (0.0)	0 (0.0)	0 (0.0)	
Negative	51 (100.0)	19 (100.0)	32 (100.0)	
Survival				
Died	128 (74.9)	58 (84.1)	70 (68.6)	**0.023** ^*^
Alive	43 (25.1)	11 (15.9)	32 (31.4)	
Mean OS (months)	11.9 ± 11.2	8.1 ± 6.1	14.4 ± 13.1	**<0.001** ^*^
Median OS (months)	9.0	7.1	12.3	

Abbreviations: ATRX, alpha‐thalassemia mental retardation X‐linked; CCRT/RT, concurrent chemoradiotherapy/radiotherapy; CT, chemotherapy; GFAP, glial fibrillary acidic protein; GTR, gross total resection; IDH1/2, isocitrate dehydrogenase 1/2; KPS, Karnofsky performance status; LMD, leptomeningeal dissemination; MGMT, O(6)‐methylguanine‐DNA methyltransferase; Olig2, oligodendrocyte transcription factor 2; OS, overall survival.

^a^ The median tumor sizes of all patients was 18.0 cm^3^. And the median tumor sizes of children and adults were17.6 cm^3^ and 18.0 cm^3^, respectively.

^*^ Bold *p* < 0.05 was considered statistically significant.

### 3.2 The survival data and prognostic factors of 171 patients with H3K27M‐mutant DMG

**FIGURE 2:**
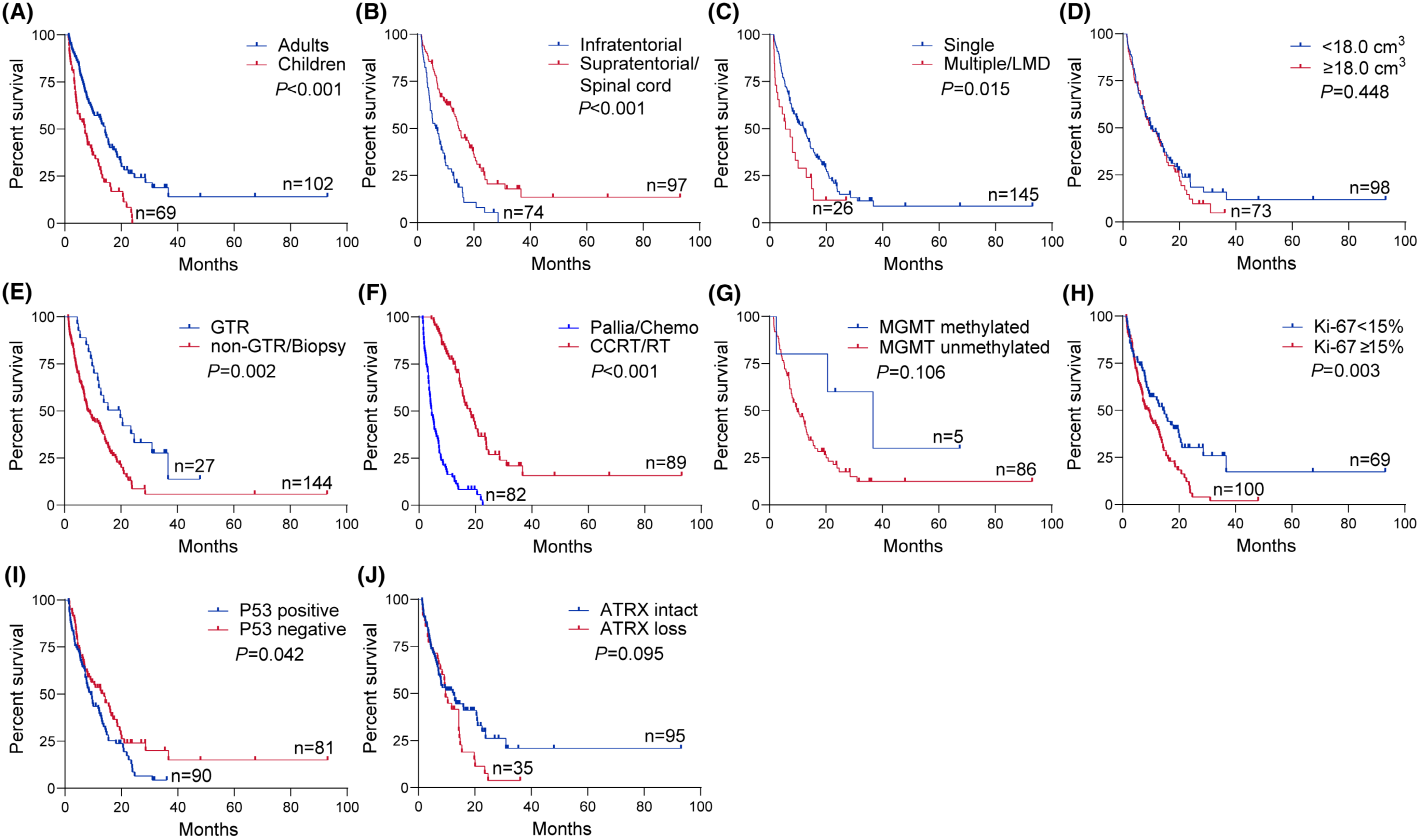
Kaplan–Meier curve of OS in the entire cohort with H3K27M‐mutant DMG. Kaplan–Meier survival curves and log‐rank test for different clinicopathological characteristics in H3K27M‐mutant DMG. Adult patients (A) had a better prognosis. Patients with tumor located in infratentorial region (B) or tumor with multiple lesions or LMD (C) were related to poorer survival time, but tumor size (D) was not significantly associated with prognosis. The extent of resection (E) and postoperative therapy (F) were relevant to prognosis, and patients undergoing GTR or CCRT/RT had a longer survival time. No statistical differences were observed in patients with *MGMT* promoter methylated and unmethylated (G). Patients with Ki‐67 index <15% (H) or *P53* non‐mutant (I) had a better prognosis. The expression of *ATRX* (J) was not related to OS. ATRX, alpha‐thalassemia mental retardation X‐linked; CCRT/RT, concurrent chemoradiotherapy/radiotherapy; Chemo, chemotherapy; GTR, gross total resection; LMD, leptomeningeal dissemination; MGMT, O(6)‐methylguanine‐DNA methyltransferase; Pallia, palliation.

The median OS of all patients was 9.0 months. The 1‐year and 2‐year OS rates for the entire cohort were 39.8% and 7.0%, respectively. Kaplan–Meier survival curves and log‐rank tests revealed that adult patients had longer survival compared to children (median OS, 12.3 vs. 7.1 months, *p* < 0.001). Additionally, it also showed adult patients (*p* < 0.001), supratentorial/spinal cord localization (*p* < 0.001), single lesion (*p* = 0.015), GTR (*p* = 0.002), CCRT/RT (*p* < 0.001), lower Ki‐67 index (*p* = 0.003), and negative P53 status (*p* = 0.042) were associated with longer OS. However, tumor size (*p* = 0.448), and other molecular markers such as *MGMT* promoter methylation (*p* = 0.106) and loss of ATRX expression (*p* = 0.095) did not show a significant effect on prognosis in the entire cohort (Figure 2).

After adjusting for confounding factors in multivariable Cox regression analysis, the results indicated pediatric patients (HR = 4.530, 95% CI: 2.058–9.970, *p* < 0.001) and loss of ATRX expression (HR = 3.131, 95% CI: 1.544–6.350, *p* = 0.002) were independent factors associated with poor survival. Conversely, single lesion (HR = 0.360, 95% CI: 0.148–0.897, *p* = 0.025) and CCRT/RT (HR = 0.134, 95% CI: 0.062–0.289, *p* < 0.001) were independent favorable prognosticators for longer overall survival in the entire cohort (Table 2).

**TABLE 2:** Multiple Cox regression analysis for OS of the entire cohort with H3K27M‐mutant DMG.

Characteristics	Hazard ratio (95% CI)	*p*‐Value
Age (children vs. adults)	4.530 (2.058–9.970)	**<0.001** ^*^
Location (infratentorial vs. supratentorial/spinal cord)	1.510 (0.773–2.949)	0.227
Spread (single vs. multiple/LMD)	0.360 (0.148–0.879)	**0.025** ^*^
Tumor volume (≥18.0 vs. <18.0 cm^3^)	1.087 (0.556–2.123)	0.808
Extent of resection (GTR vs. non‐GTR/Biopsy)	2.000 (0.747–5.354)	0.167
Postoperative therapy (CCRT/RT vs. palliation/chemotherapy)	0.134 (0.062–0.289)	**<0.001** ^*^
*MGMT* promoter (unmethylated vs. methylated)	1.609 (0.253–10.225)	0.614
Ki‐67 index (≥15% vs. <15%)	1.098 (0.503–2.397)	0.815
P53 (Negative vs. Positive)	1.440 (0.691–3.002)	0.330
ATRX (Loss vs. Intact)	3.131 (1.544–6.350)	**0.002** ^*^

*Note*: The multivariable Cox regression analysis was performed based on all variables in the univariate analysis.

Abbreviations: ATRX, alpha‐thalassemia mental retardation X‐linked; CCRT/RT, concurrent chemoradiotherapy/radiotherapy; GTR, gross total resection; LMD, leptomeningeal dissemination; MGMT, O(6)‐methylguanine‐DNA methyltransferase.

^*^ Bold *p* < 0.05 was considered statistically significant.

### 3.3 The survival data and specific prognostic factors in adult and children DMG subgroups

**FIGURE 3:**
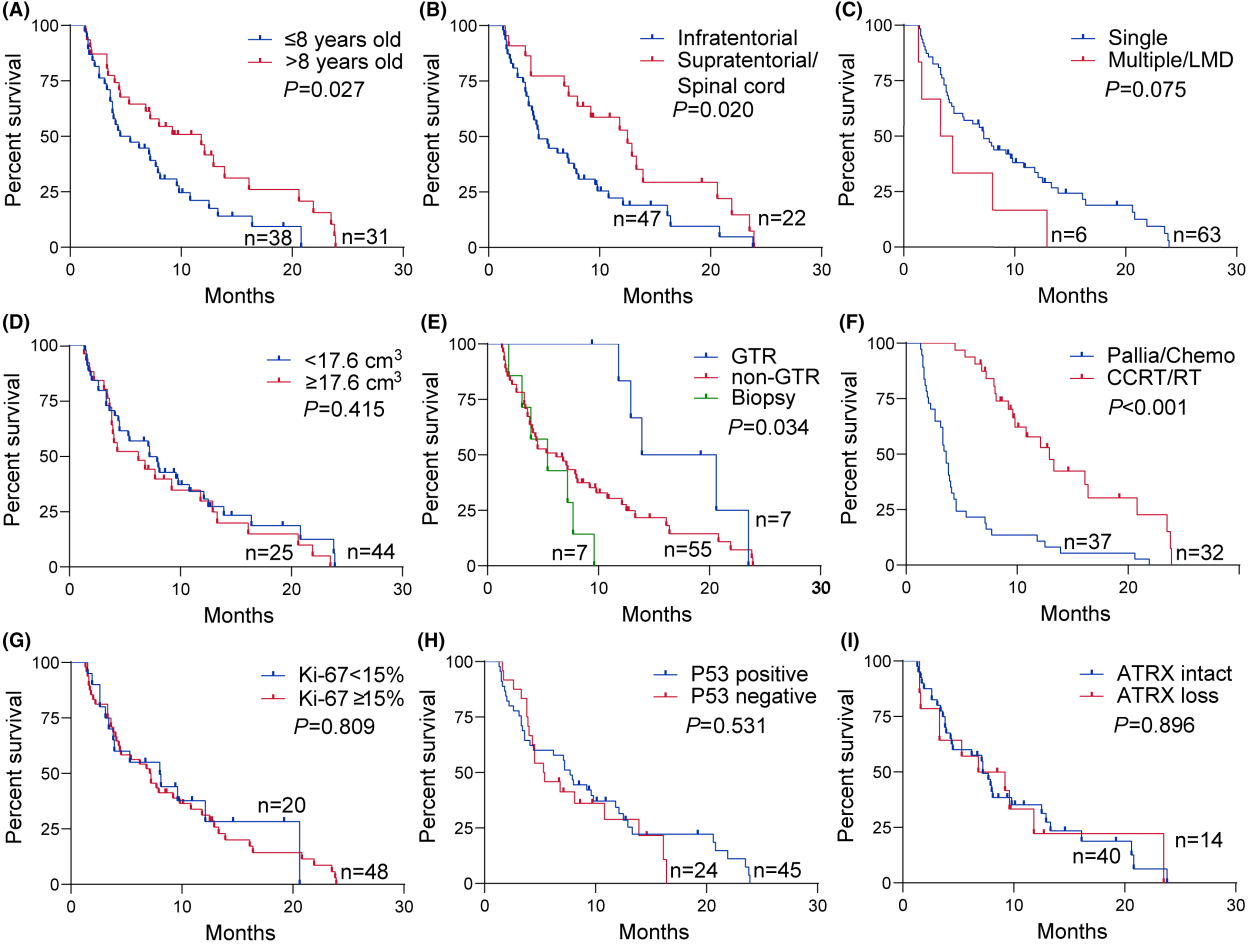
Kaplan–Meier curve of OS in pediatric patients with H3K27M‐mutant DMG. Kaplan–Meier survival curves and log‐rank test for different clinicopathological characteristics in pediatric patients with H3K27M‐mutant DMG. Pediatric patients with age more than 8 years old (A) had a longer survival OS. Tumor located in infratentorial region (B) was associated with poorer prognosis, but tumor spread (C) and tumor size (D) were not significantly related to prognosis. The extent of resection (E) and postoperative therapy (F) were associated with survival time, and patients undergoing GTR or CCRT/RT had a longer OS. The molecular alterations or expression containing Ki‐67 (G), *P53* (H), and *ATRX* (I) were not relevant to prognosis of pediatric patients. ATRX, alpha‐thalassemia mental retardation X‐linked; CCRT/RT, concurrent chemoradiotherapy/radiotherapy; Chemo, chemotherapy; GTR, gross total resection; LMD, leptomeningeal dissemination; Pallia, palliation.

**FIGURE 4:**
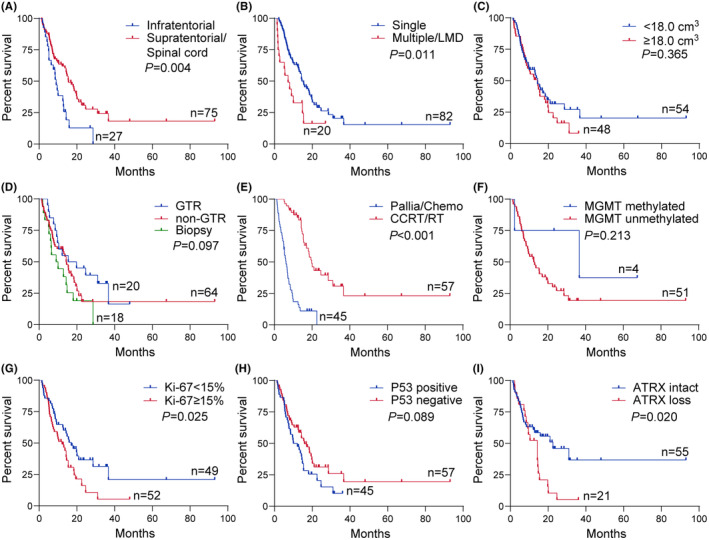
Kaplan–Meier curve of OS in adult patients with H3K27M‐mutant DMG. Kaplan–Meier survival curves and log‐rank test for different clinicopathological characteristics in adult patients with H3K27M‐mutant DMG. Adult patients with tumor occurred in infratentorial region (A) or tumor with multiple lesions or LMD (B) were relevant to poorer OS, but tumor size (C) was not associated with prognosis. No statistically significant differences were found in the extent of resection (D). The postoperative therapy (E) was associated with OS, and patients undergoing CCRT/RT had a better prognosis. *MGMT* promoter methylated and unmethylated (F) was not related to OS. Adult patients with Ki‐67 index <15% (G) had a longer survival time. Expression of P53 (H) was not significantly relevant to prognosis. *ATRX* intact expression (I) was associated with longer OS. ATRX, alpha‐thalassemia mental retardation X‐linked; CCRT/RT, concurrent chemoradiotherapy/radiotherapy; Chemo, chemotherapy; GTR, gross total resection; LMD, leptomeningeal dissemination; MGMT, O(6)‐methylguanine‐DNA methyltransferase; Pallia, palliation.

We conducted separate survival analyses for each subgroup to examine the prognostic factors specific to DMG in children and adults. Similar with the entire cohort, CCRT/RT and supratentorial/spinal cord localization were associated with longer survival in both pediatric and adult subgroups. In the adult DMG patients, single lesion (*p* = 0.011) and intact ATRX expression (*p* = 0.020) were significantly associated with a better prognosis. In the pediatric patients, GTR (*p* = 0.034) was associated with better survival. Specifically, children aged 8 years or younger had even worse survival (*p* = 0.027) (Figures 3 and 4).

Cox proportional hazard model confirmed that CCRT/RT was an independent favorable prognostic factor for both pediatric (HR = 0.127, 95% CI: 0.064–0.250, *p* < 0.001) and adult (HR = 0.120, 95% CI: 0.060–0.240, *p* < 0.001) subgroups. Additionally, infratentorial tumors (HR = 4.022, 95% CI: 1.997–8.101, *p* < 0.001) associated with poor survival in pediatric DMG patients. In contrast, in adult subgroup, single lesion (HR = 0.365, 95% CI: 0.179–0.746, *p* = 0.006) was an independent favorable factor, while loss of ATRX expression (HR = 2.214, 95% CI: 1.194–4.106, *p* = 0.012) was an independent adverse factor in adult patients (Tables 3 and 4).

**TABLE 3:** Multiple Cox regression analysis for OS of pediatric patients with H3K27M‐mutant DMG.

Characteristics	Hazard ratio (95% CI)	*p*‐Value
Location (infratentorial vs. supratentorial/spinal cord)	4.022 (1.997–8.101)	**<0.001** ^*^
Therapy (CCRT/RT vs. palliation/chemotherapy)	0.127 (0.064–0.250)	**<0.001** ^*^

*Note*: The multivariable Cox regression analysis was performed based on variables with *p* < 0.05 in the univariate analysis.

Abbreviation: CCRT/RT, concurrent chemoradiotherapy/radiotherapy.

^*^ Bold *p* < 0.05 was considered statistically significant.

**TABLE 4:** Multiple Cox regression analysis for OS of adult patients with H3K27M‐mutant DMG.

Characteristics	Hazard ratio(95% CI)	*p*‐Value
Spread (single vs. multiple/LMD)	0.365 (0.179–0.746)	**0.006** ^*^
Therapy (CCRT/RT vs. palliation/chemotherapy)	0.120 (0.060–0.240)	**<0.001** ^*^
*ATRX* (loss vs. intact)	2.214 (1.194–4.106)	**0.012** ^*^

*Note*: The multivariable Cox regression analysis was performed based on variables with *p* < 0.05 in the univariate analysis.

Abbreviations: ATRX, alpha‐thalassemia mental retardation X‐linked; CCRT/RT, concurrent chemoradiotherapy/radiotherapy; LMD, leptomeningeal dissemination.

^*^ Bold *p* < 0.05 was considered statistically significant.

## 4 DISCUSSION

H3K27M‐mutant DMG is an aggressive malignancy with a dismal prognosis. Since its introduction in 2016 and low incidence rate, there have been limited large cohort studies on its biological behavior, optimal treatment, and molecular characteristics associated with prognosis. The clinicopathological characteristics of pediatric DMG have been relatively well uncovered, whereas few studies have compared pediatric and adult subgroups, and some of their results were not consensus. Our study presented the clinical, histopathologic, and genetic features of 171 patients with H3K27M‐mutant DMG stratified by age. Meanwhile, we explored the prognostic factors of patients in the entire cohort and pediatric and adult subgroups.

In this large cohort, the mean age was 27.0 ± 18.3 years, with a higher proportion of adults compared with children, which was consistent with the demographic characteristic of no predilection on age in recent studies without age limit on patient selection,^5^, ^6^, ^28^, ^31^, ^32^ implying that H3K27M detection was not ignored in adult midline tumors. A series of retrospective studies or systematic reviews revealed that patients with thalamic or spinal tumors are older than those with brainstem tumors.^7^, ^33^, ^34^ Similar to these studies, we found that pediatric patients frequently occurred in the infratentorial region, and adult patients were commonly located in the supratentorial region. Besides, the proportion of adults with spinal cord tumors was higher than that of children. In our study, most patients exhibited a single lesion, and a minority exhibited multiple lesions or LMD. Multiple lesions more frequently occurred in adults than in children. Previous studies reported LMD occurred in DIPG but rarely in supratentorial DMG, indicating that the leptomeningeal spreading behavior of DMG differs from that of other high‐grade gliomas.^28^, ^35^, ^36^, ^37^, ^38^, ^39^ Moreover, higher expression of Ki‐67 and positive P53 status were commonly observed in children compared with adults, which is consistent with previous studies.^26^, ^33^

When stratified by age, the median OS for adults was 12.3 months, longer than the 7.1 months observed in children. Cox regression analysis revealed that pediatric patient was an unfavorable independent factor in the whole cohort. Most retrospective studies have demonstrated that the prognosis for adult patients was superior to that of historical pediatric survival.^40^, ^41^, ^42^ A retrospective cohort study with 164 H3K27M‐mutant DMG patients revealed that age was an independent prognostic factor.^26^ The same result was also found in a systematic review.^34^ However, a small‐sized study and a study on brainstem glioma revealed no significant difference in prognosis between adults and children.^28^, ^29^ The distinct mechanisms accounting for the different prognosis between children and adults have not been fully explored. A systematic review including 43 studies showed more aggressive features, including higher rates of pathologic features of high‐grade tumors and a higher Ki‐67 proliferation index, occurred in pediatric H3K27M‐mutant DMG when compared to adults, which resulted in a worse prognosis in the children subgroup.^25^ Moreover, there are differences in the molecular characteristics of H3K27M‐mutant DMG between pediatric and adult patients. Several studies demonstrated that *ACVR1* mutation frequently occurred in children, whereas *FGFR1*, neurofibromatosis type 1 (*NF1*), and *ATRX* mutations were more common in adults.^25^, ^26^ The differential alteration of these molecules may be related to tumor malignancy, and further studies are required to explore the molecular mechanisms in H3K27M‐mutant DMG, especially in different age subgroups.

Furthermore, in the pediatric subgroup, we found that children aged ≤8 years had a shorter OS. Several previous studies have also investigated the prognosis of pediatric DIPG in different age subgroups. An earlier study with 316 pediatric patients showed that age ≤3 years positively predicted the prognosis of DIPG.^43^ Moreover, an international cohort demonstrated that DIPG patients aged ≤36 months had increased long‐term survival,^44^ and suggested that patients of different ages could have different prognoses, especially within the pediatric group. A recent study profiled the landscape of cell states and tumor microenvironment, which were found to be different in H3K27M‐mutant DMG between the pediatric and adult patients,^45^ probably accounting for the different prognoses. In the future, the prognosis of patients with DMG should be further analyzed in age stratification as a continuous variable.

Recently, some retrospective studies have revealed that DMG in different locations may exhibit distinct outcomes, and H3K27M‐mutant DMG tumors in the infratentorial region indicated poorer survival than those in the supratentorial region.^26^, ^34^, ^46^, ^47^ In this study, the patients with supratentorial/spinal cord tumors exhibited a longer survival time, and tumor location was an independent prognostic factor in the pediatric cohort, but not in adults. Other studies also demonstrated that tumor location was not statistically relevant to the survival of adult DMG.^48^, ^49^

Single lesion was another favorable prognosticator in the entire cohort and the adult subgroup when compared with multiple lesions or LMD. The prognostic value of dissemination characteristics was rarely reported. LMD was related to shorter progression‐free survival in DIPG.^35^ A recent study involving 42 pediatric patients with high‐grade midline thalamic gliomas revealed a correlation between LMD and a poor survival outcome.^50^ In our study, tumor spread was not statistically relevant with prognosis in pediatric patients; this may be attributed to the limited numbers of pediatric patients with multiple lesions/LMD.

Surgical resection is one of the standard therapeutic approaches in H3K27M‐mutant DMG. However, due to the deep location and diffused and infiltrative behavior of DMG, total resection is usually impractical, especially in the brainstem. Moreover, unlike glioblastoma, the benefit from surgery in DMG is unconfirmed. Most experts advocate maximal resection or even extended resection for glioblastoma because the extent of resection is unquestionably associated with prognosis.^51^, ^52^ Multivariate Cox regression analysis in our study revealed that GTR could not independently improve OS in the entire cohort or the pediatric/adult subgroups. Consistent with our study, other cohorts also found that extent of resection was not a prognosticator in DMG.^46^, ^51^, ^53^ However, the multivariate Cox regression analysis of a systematic review with 484 patients revealed that resection was associated with a better prognosis than biopsy in H3K27M‐mutant DMG.^34^

According to guidelines and consensus, radiotherapy is recommended as a standard and efficient strategy for DMG, as it improves the patient's quality of life and prognosis.^15^ After standard radiotherapy, 70%–80% of patients experience temporary symptom relief, and the survival rate is increased.^16^, ^17^, ^18^ The results also indicate that postoperative CCRT/RT significantly prolongs survival time in the entire cohort. However, since most patients in our study received CCRT, we could not differentiate the benefit from concurrent TMZ. Previous studies have demonstrated that adding TMZ to radiotherapy does not provide additional benefits due to the intact blood–brain barrier and the specific molecular alterations in most cases with MGMT promoter unmethylation.^19^, ^20^, ^21^, ^54^ Besides, various chemoresistance mechanisms including genetic alterations, DNA double‐strand breaks repair, glioma stem cells, and tumor suppressor microenvironment also limit the efficacy of chemotherapy in glioma patients.^55^ Our findings are consistent with the long‐standing view that chemotherapy or targeted therapy without radiotherapy offers limited survival benefits for DIPG or DMG in several clinical trials.^56^, ^57^, ^58^, ^59^ A meta‐analysis also revealed that no chemotherapeutic treatment showed benefits over conventional radiotherapy.^60^

Moreover, CCRT/RT was identified as a favorable prognostic factor both in pediatric and adult patients. Similarly, several retrospective studies focusing on either adults or children have shown that postoperative adjuvant treatments are associated with improved prognosis. A study with 96 adult DIPG patients found that postoperative treatment was an important predictor for H3K27M‐mutant tumors, especially in patients with a symptom duration of ≤4 months.^61^ Furthermore, in pediatric DIPG, a single‐center study with 50 patients demonstrated that patients receiving combination treatment in addition to radiotherapy or chemotherapy alone had a better prognosis.^62^ Some studies have also indicated that radio‐chemotherapy may be more effective in adult DIPG patients.^61^, ^63^, ^64^ However, a cohort study with 38 adult patients with thalamic DMG found no statistical difference in survival between radiotherapy plus chemotherapy and chemotherapy alone.^65^

Recently, molecular characteristics have been recognized as predictive factors in gliomas. *P53* mutation is frequently observed in H3K27M‐mutant DMG,^8^, ^66^ and *P53* overexpression or mutation has been identified as an independent risk factor for shorter OS in H3K27M‐mutant DMG.^32^, ^67^ Our Kaplan–Meier survival analysis revealed that P53 mutation was associated with shorter survival time in the entire cohort. Higher Ki‐67 index has been correlated with shorter survival in H3K27M‐mutant DMG.^68^ Similarly, our results showed that Ki‐67 index was associated with survival in the entire cohort and adult patients, although it was not an independent prognostic factor for them. Studies have also indicated that *ACVR1* and *FGFR1* mutations and *PDGFRA* amplification are related to the prognosis of H3K27M‐mutant DMG.^8^, ^9^ Picca et al.^69^ found that telomerase reverse transcriptase (*TERT*) mutation, cyclin‐dependent kinase inhibitor 2A (*CDKN2A*) deletion, and *EGFR* amplification were correlated with worse prognosis in adult midline gliomas. Our study identified loss of ATRX expression as an unfavorable prognosticator in the entire cohort and adult subgroup. *ATRX* mutation is observed in various gliomas, particularly in low‐grade gliomas with *IDH* mutation.^70^ *ATRX* deficiency impairs non‐homologous end joining (NHEJ) by hindering the recruitment of pDNA‐PKcs, potentially through conformational changes in heterochromatin, leading to genetically unstable tumors and aggressive tumor behavior.^71^ The function of *ATRX* loss in H3K27M‐mutant DMG remains unknown, but studies have suggested that *ATRX* loss played an essential role in DMG development.^71^ Additionally, loss of *ATRX* induces an immunosuppressive microenvironment in gliomas by promoting the secretion of immunosuppressive cytokines and upregulating the expression of immune checkpoint molecules, suppressing anti‐tumor immunity.^72^ A cohort study with 43 cases of H3K27M‐mutant DMG found that *ATRX* loss was associated with shorter overall survival,^32^ which is consistent with our results. However, other studies have shown that the patients with *ATRX* loss have significantly longer survival compared to those with *ATRX* intact expression in diffuse gliomas.^8^, ^26^, ^73^ Further studies are needed to explore the mechanism of *ATRX* loss in H3K27M‐mutant DMG and its implications in different age populations.

There are limitations to this study. Firstly, since most of the patients (n = 153) were enrolled before June 2021, we did not include those who harbored EZHIP overexpression, which also results in a global reduction of H3K27me3 according to the 5th edition of the WHO Classification of CNS Tumors. Thus, instead of the new term “diffuse midline glioma, H3 K27‐altered,” we still use the term “diffuse midline glioma, H3 K27‐mutated.” In future studies, we plan to adopt the newest diagnostic criteria to examine EZHIP and H3K27me3 expression. Secondly, we did not acquire comprehensive genetic information on the patients. Therefore, the next steps in our study are to collect tumor tissue samples and explore the transcriptome and genetic alterations of H3K27M‐mutant DMG using RNA‐sequencing and next‐generation sequencing. Moreover, this retrospective study has inherent selection bias, and the sample size is not large enough for robust subgroup analysis.

## 5 CONCLUSION

This study provided a comprehensive analysis of the clinicopathological and genetic features of the entire population and stratified them by age in H3K27M‐mutant DMG. We have identified postoperative concurrent chemoradiotherapy/radiotherapy as a significant independent prognostic factor for both the entire cohort and pediatric/adult subgroups. Additionally, we have made innovative findings, including the identification of single lesion and ATRX intact expression as independent favorable prognostic factors in adults. We have also observed that tumor location significantly impacts survival of pediatric patients, and children below the age of 8 years tended to have even poorer outcomes. These results suggest that the clinical outcomes of H3K27M‐mutant DMG should be stratified based on molecular characteristics and age. This finding can aid neuro‐oncologists in risk stratification and selecting individualized treatment options.

## AUTHOR CONTRIBUTIONS

Conception and design of the study: CL. Statistics analysis: XG, SK, and CL. Manuscript drafting: SK, XG, and CL. Acquisition of data: DD, JW, and LZ. All authors have read and agreed to the published version of the manuscript.

## FUNDING INFORMATION

This study was supported by the National Natural Science Foundation of China (Grant No. 81701285), the Natural Science Foundation of Hunan Province (Grant No. 2018JJ3824), and the Nature Science Youth Foundation of Hunan Province (Grant No. 2018JJ3856).

## CONFLICT OF INTEREST STATEMENT

The authors declare no conflict of interest.

## Data Availability

The data supporting the conclusion of this article will be made available from the corresponding authors upon on reasonable request.
